# Association of Continuity of Primary Care and Statin Adherence

**DOI:** 10.1371/journal.pone.0140008

**Published:** 2015-10-08

**Authors:** James R. Warren, Michael O. Falster, Bich Tran, Louisa Jorm

**Affiliations:** 1 Department of Computer Science, University of Auckland, Auckland, New Zealand; 2 Centre for Big Data Research in Health, University of New South Wales, Kensington, New South Wales, Australia; University of Nevada Las Vegas, UNITED STATES

## Abstract

**Purpose:**

Deficiencies in medication adherence are a major barrier to effectiveness of chronic condition management. Continuity of primary care may promote adherence. We assessed the association of continuity of primary care with adherence to long-term medication as exemplified by statins.

**Research Design:**

We linked data from a prospective study of 267,091 Australians aged 45 years and over to national data sets on prescription reimbursements, general practice claims, hospitalisations and deaths. For participants having a statin dispense within 90 days of study entry, we computed medication possession ratio (MPR) and usual provider continuity index (UPI) for the subsequent two years. We used multivariate Poisson regression to calculate the relative risk (RR) and 95% confidence interval (CI) for the association between tertiles of UPI and MPR adjusted for socio-demographic and health-related patient factors, including age, gender, remoteness of residence, smoking, alcohol intake, fruit and vegetable intake, physical activity, prior heart disease and speaking a language other than English at home. We performed a comparison approach using propensity score matching on a subset of the sample.

**Results:**

36,144 participants were eligible and included in the analysis among whom 58% had UPI greater than 75%. UPI was significantly associated with 5% increased MPR for statin adherence (95% CI 1.04–1.06) for highest versus lowest tertile. Dichotomised analysis using a cut-off of UPI at 75% showed a similar effect size. The association between UPI and statin adherence was independent of socio-demographic and health-related factors. Stratification analyses further showed a stronger association among those who were new to statins (RR 1.33, 95% CI 1.15–1.54).

**Conclusions:**

Greater continuity of care has a positive association with medication adherence for statins which is independent of socio-demographic and health-related factors.

## Introduction

Poor adherence (also known as *compliance*) to long-term medication is a major issue undermining effective delivery of healthcare.[1] It is frequently overlooked by prescribing physicians when intensifying treatment.[2, 3] Statins, as a case in point, are effective in primary prevention of cardiovascular disease (CVD)[4] and are a central element of CVD risk management guidelines.[5]^,^[6] The rate of failure to maintain statin therapy for 12 months after initiation is high,[7] even following acute coronary events.[8] Poorer levels of statin adherence are associated with higher rates of long-term mortality after acute myocardial infarction[9] and in coronary artery disease generally.[10] Risk factors for poor adherence to statins include dispensing for primary (as compared to secondary) prevention[11, 12] and being a new statin user.[11] In terms of strategies to improve adherence to lipid lowering drugs, reinforcement and reminder have the best evidence.[13]

The relationship of continuity of care (CoC) to medication, including statin, adherence is unclear. Brookhart et al.[14] found that physician visits–either to the physician who initiated statin therapy, or to another physician–as well as cholesterol tests, myocardial infarction or other CVD-related hospitalisation, were all associated with return to statin adherence. Adding to the complexity, there is a ‘healthy user bias’ in statin adherence; that is, those who adhere to statins tend to pursue other healthy practices, including seeking out preventative health services in the form of screening tests and vaccinations,[15] and being more likely to be non-smokers.[12, 16]

The present study utilised data from a large prospective study of Australians aged 45 and over linked with national health databases to estimate the association of CoC on statin adherence when adjusting for a range of patient characteristics.

## Methods

### Data sources

The 45 and Up Study is a cohort study of more than 260,000 men and women aged 45 years and over resident in New South Wales (NSW), Australia; managed by the Sax Institute, it is an open research resource to help facilitate research on health, ageing and quality-of-life.[17] Participants for the 45 and Up Study were randomly sampled from the enrolment database of Medicare Australia (Australia’s universal health insurance scheme) and joined the study by completing a mailed self-administered questionnaire and providing written informed consent for participation and long term follow-up, including linkage to health records. The response rate was 18%.[17] Recruitment to the 45 and Up Study commenced in 2005 and was completed in 2009. The data set for the present analysis was created by linkage of 45 and Up Study baseline survey data to Australian Government and NSW state data sources as described below.

Through the Pharmaceutical Benefits Scheme (PBS), the Australian Government subsidises essential medications[18] including statins. The PBS data provides a transaction record for each subsidised dispense from a community pharmacy. Concession Card holders have a lower subsidy co-payment threshold than general beneficiaries, and there is an annual Safety Net threshold of total family payments after which prescriptions are fully subsidised by the PBS.[19] Concession Card holder status for PBS is granted for people aged 65 and over who meet an income test, as well as for disability, low income or facing a large burden of dependants.[20] The PBS data includes the recipient’s Concession Card status and whether the Safety Net threshold had been reached at the time of the transaction.

The Medical Benefits Schedule (MBS) is Australia’s universal health insurance scheme for subsidised medical care including general consultations, diagnostic tests and pathology services. Only services attracting subsidy benefit are included in this database.

The NSW Admitted Patient Data Collection (APDC) is a routinely collected census of hospital separations (discharges, transfers and deaths) from all NSW public and private sector hospitals and day procedure centres.

The NSW Registry of Births, Deaths and Marriages (RBDM) data captures details of all deaths registered in NSW.

The Sax Institute linked 45 and Up Study questionnaire data and MBS and PBS claims using a scrambled Medicare number, while the Centre for Health Record Linkage (www.cherel.org.au) performed linkage to the APDC and RBDM data using probabilistic methods and commercial software (ChoiceMaker; ChoiceMaker Technologies Inc.). Quality assurance data show false positive and negative rates for data linkage of 0.4% and less than 0.1%, respectively.

### Participants and exclusion criteria

Using the linked data set, we identified 45 and Up Study participants who had (a) entered the 45 and Up Study (completed the baseline survey) between 1 July 2006 and 30 June 2009; and (b) had a statin dispensed within 90 days of study entry. For these, we defined an evaluation period (EP) of two years starting from study entry.

**Fig 1:**
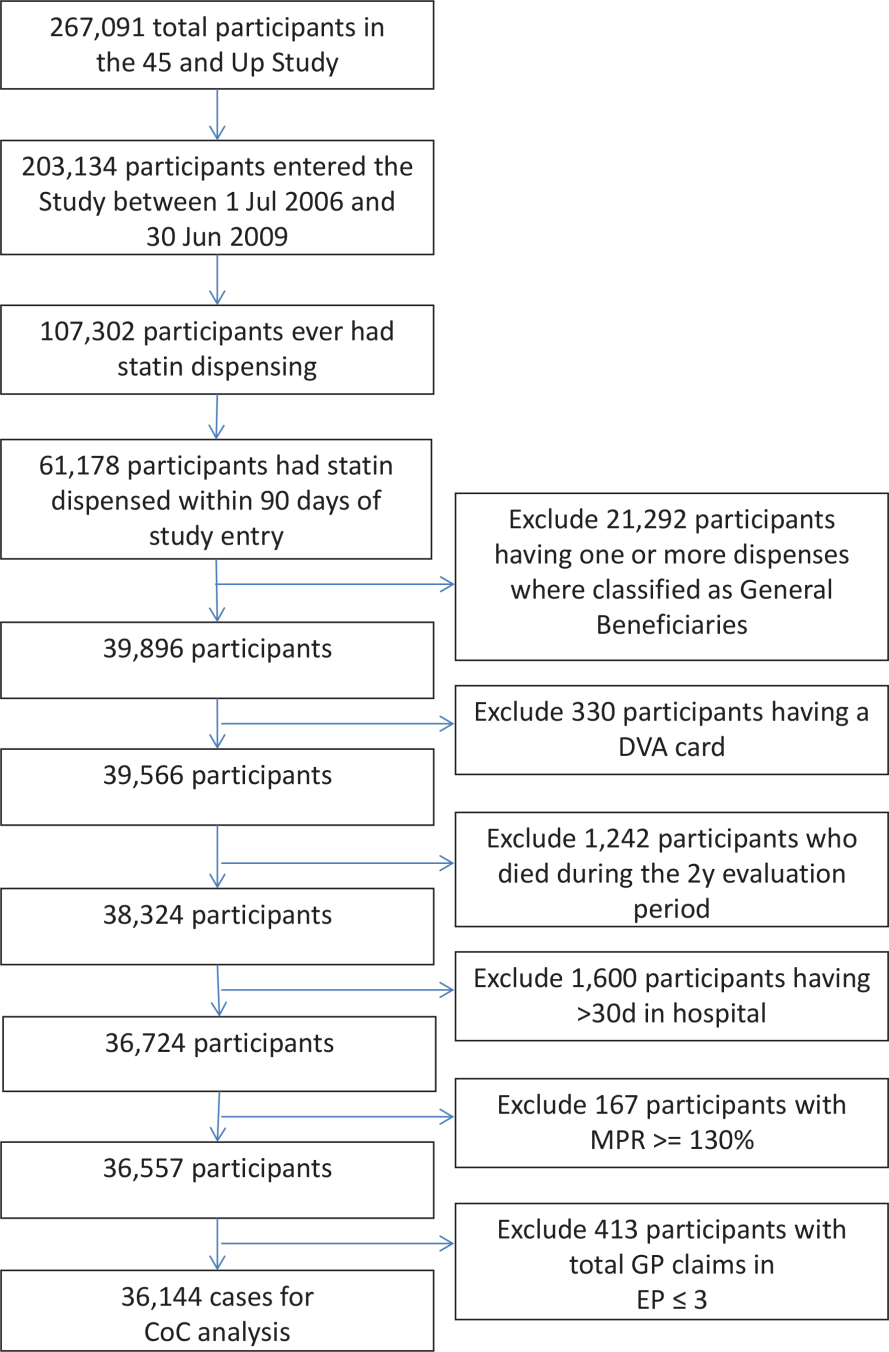
Participant case selection flowchart.

We excluded participants for whom we expected data capture in PBS claims data to be less accurate. As such, we excluded those with any PBS transactions showing patient type “General–Ordinary” or “General–Safety Net” during the EP as many common statins fall under the co-payment threshold for general beneficiaries and would not have been captured[21] (conversely, all statin dispensing for Concession Card holders would be subsidized and thus a complete record of community based supply is expected during the EP). We also excluded participants who held a Department of Veterans Affairs (DVA) healthcare card because Medicare data do not capture all services provided to these cardholders. We excluded participants who died during the EP as indicated by an RBDM record. We also excluded participants who had >30 days in hospital during the EP as they might have received statin therapy as an inpatient that was not captured by the PBS. Further exclusions were applied for participants with unusually high levels of medication supply and for participants with too few GP claims in the EP to ensure the stable estimation of measures of CoC as described, respectively, in the ‘Outcome’ and ‘Measures of continuity of care’ sections below. A flowchart describing the inclusion and exclusion of participants is shown in Fig 1.

### Outcome

We assessed statin adherence in terms of a medication possession ratio (MPR), computed as the proportion of days covered by dispensing to the patient as indicated from the PBS records during the two-year EP. We took the commonly used threshold of MPR`80% to indicate adherence.[22] Statins at all common strengths are packaged for 30-days’ supply per dispense; thus, we defined MPR as the number of PBS records for statins (as per ATC[23] codes, including combination products) with dispense dates within that participant’s EP divided by 24. We excluded anomalous cases showing MPR ≥ 130% as this represents more than 7 months over-supply and is difficult to justify in terms of expected fluctuations in supply pattern (e.g. as with a dose change).

### Measures of continuity of care

We assessed continuity of care using all MBS claims categorised as ‘A1’ and ‘A2’ (general practitioner attendances and other non-referred attendances to which no other item applies)[24] during the EP, on the basis that while consultant (specialist) physicians may be involved in commencement or adjustment of therapy, General Practitioners (GPs) are the usual providers of statin therapy in the Australian healthcare system.

We defined continuity of care in two ways: firstly, using the usual provider continuity index (UPI),[25] which measures the concentration of a patient’s total visits to the most common provider of care; and secondly, using the ‘continuity of care score’ (CoC score),[26] which measures concentration of patient visits across providers. UPI and CoC scores were calculated using the following formulae:

$$\text{UPI}=\frac{\max({\text{n}}_{i})}{\text{N}}$$

$$\text{CoC Score}=\frac{\sum {{\text{n}}_{i}}^{2}-\text{N}}{\text{N}(\text{N}-1)}$$

where n_*i*_ = number of visits that the participant has with their *i*th provider, max(n_*i*_) = number of visits the patient has with the provider with whom they have the most visits, and N = total visits. UPI and CoC scores are expected to be highly correlated[27] and accordingly we selected UPI as the primary focus for our analysis, retaining CoC score for sensitivity analysis only. We defined tertiles of UPI and CoC score and repeated the analysis using dichotomised cut-off of UPI at 75%.[28]

Analyses were restricted to participants with 4 or more GP claims in the EP as there is limited granularity in possible continuity scores with a smaller number of visits.[29]

### Statistical analyses

Given the common outcome, we used modified Poisson regression [30, 31] to estimate relative risks (RRs) and 95% confidence intervals (CIs) for the association between CoC and MPR for statin adherence. Covariates were extracted from the self-reported questionnaire in the 45 and Up Study, except for new-to-statins status which we defined as no PBS record of statin dispensing in the 24- month period prior to the EP. We built two sequential regression models: model 1, adjusted for age and gender only; and model 2, adjusted for age, gender and a range of other socio-demographic and health-related variables. All covariates are described in Table 1. We conducted a series of interaction analyses between UPI and each of the covariates and performed stratified analyses only for the covariates that showed significant interaction (p<0.05).

**Table 1:** Model predictors of adherence.

Model	Variable	Description
**Model 1 and model 2**	Continuity of care	Usual Provider Continuity Score (UPI)[25] (tertile and dichotomous ≥ 75%, proportion of visits to most-visited provider); alternative: CoC score[26], concentration of patient visits across set of providers
	Age	At time of survey completion (July 1, 2006 –June 30, 2009)
	Gender	As per Australian Medicare profile (which can be updated by the individual)
**Model 2 only**	Highest Education Qualification	Self-reported
	Aboriginal or Torres Strait Islander	Self-reported
	Language other than English	Language spoken at home
	Partnership Status	Marriage or partner versus never married, separated, divorced or widowed
	Private Health Insurance	Self-report of private insurance (at levels of basic private hospital cover or ‘with extras,’ indicating additional cover for ancillary non-hospital services), or Health Care Card[20]
	Employment status	Including self-employed
	Annual income	Self-reported
	ARIA+ Remoteness	Accessibility and Remoteness Index for Australia Plus (ARIA+) score for the postcode of residential address*
	Body Mass Index	From self-reported height and weight†
	Current Smoking Status	Self-reported
	Alcohol Drinks / week	Self-reported
	Sufficient Fruit and Vegetables	≥ 2 servings per day of fruit and 5 of vegetables
	Sufficient Physical Activity	At least 150 MET (Metabolic Equivalent Task) adjusted minutes over 5 sessions per week
	Self-Rated Health	Self-reported “Overall health”
	New to Statins	Considered ‘new’ to statin therapy if there are no PBS records of statins dispensed in the 2 years prior to the study entry date
	Self-Reported Heart Disease	Response to “Has a doctor ever told you that you have any of the following…” with tick-box for Heart Disease
	Comorbidities	Number of self-reported conditions, out of heart disease, high blood pressure, stroke, diabetes, blood clot, asthma, Parkinsons disease, and any cancer except skin cancer
	Functional Limitations	Medical Outcomes Study Physical Functioning (MOSPF) scale[32]
	Psychological Distress	Kessler–10 (K10) score[33]

* ARIA+ is based on sum of ratios of road distances to population centers of five distinct sizes as compared to Australian national averages[34]. We label ARIA+ bands: 0–1.84 = Metro; >1.84–3.51 = Inner Regional; >3.51–5.80 = Outer Regional; >5.80–9.08 = Remote; and >9.08 = Very Remote.

† BMI categories are labelled conventionally as Underweight (BMI<20), Normal weight (BMI 20 –<25), Overweight (BMI 25 –<30) and Obese (BMI 30 and higher).

We used propensity score matching as a sensitivity analysis to control for confounding. We estimated the probability of being in each level of UPI (propensity score) using logistic regression conditioning on all variables listed in Table 1 and used a greedy matching technique[35] to identify matched pairs of participants (1:1) with similar propensity scores. We used conditional logistic regression to estimate the effect size of UPI on MPR in the matched subsets of participants.

We carried out all analyses in SAS 9.3 (SAS Institute, Cary, NC).

The NSW Population & Health Services Research Ethics Committee (reference 2011/12/362), the University of Western Sydney Ethics Committee (reference H9517), and the Aboriginal Health & Medical Research Ethics Committee (reference 832/11) approved the research. The University of New South Wales Human Research Ethics Committee approved the 45 and Up Study.

## Results

We included for analysis 36,144 (13.5%) of the 267,091 participants in the 45 and Up Study after exclusions as shown in Fig 1. Table 2 shows the ranges, statin adherence rates, and multivariate adjusted RR with 95% CI for statin adherence for UPI tertiles, dichotomised UPI and CoC score tertiles with the two sets of covariates as per Table 1. The tertile cut-offs of UPI were at 68.7% and 88.9%; 15,179 participants (42%) had a UPI < 75%. Compared to the lowest tertile, the upper tertile of UPI was associated with a 5% increased MPR for statin adherence (CI 1.04–1.06). Dichotomised analysis showed that UPI at 75% or above was also associated with increased likelihood of adherence (RR 1.04, 95% CI 1.03–1.05). The effect sizes of UPI on MPR were independent of socio-demographic and health factors with model 1 and model 2 returning nearly-identical results. As expected, UPI and CoC score were highly correlated, Pearson correlation coefficient = 0.977; RRs by tertile were almost identical for UPI and CoC score.

**Table 2:** Relative Risk (RR) of continuity of care, measured by the Usual Provider Continuity Index (UPI) and the Continuity of Care score (CoC score), on statin adherence (Medication Possession Ratio, MPR ≥ 80%) in models adjusted for covariates as per Table 1.

		Statin adherence (MPR ≥ 80)		Adjusted RR (95% CI)	
	Range of continuity measure (min-max)	No	Yes	Model 1	Model 2
**UPI tertiles**					
Low	9.0–66.7	2768 (22.8)	9354 (77.2)	**1.00**	**1.00**
Medium	66.8–88.9	2324 (19.1)	9825 (80.9)	**1.04 (1.03–1.06)**	**1.04 (1.02–1.05)**
High	89.0–100	2163 (18.2)	9710 (81.8)	**1.05 (1.04–1.07)**	**1.05 (1.04–1.06)**
**UPI ≥0.75**					
No	9.0–74.9	3354 (22.1)	11825 (77.9)	**1.00**	**1.00**
Yes	75–100	3901 (18.6)	17064 (81.4)	**1.04 (1.03–1.05)**	**1.04 (1.03–1.05)**
**CoC score tertiles**					
Low	0–48.5	2762 (22.9)	9290 (77.1)	**1.00**	**1.00**
Medium	48.5–78.6	2298 (19.1)	9749 (80.9)	**1.04 (1.03–1.06)**	**1.04 (1.03–1.05)**
High	78.6–100	2195 (18.2)	9850 (81.8)	**1.05 (1.04–1.07)**	**1.05 (1.04–1.07)**

**Fig 2:**
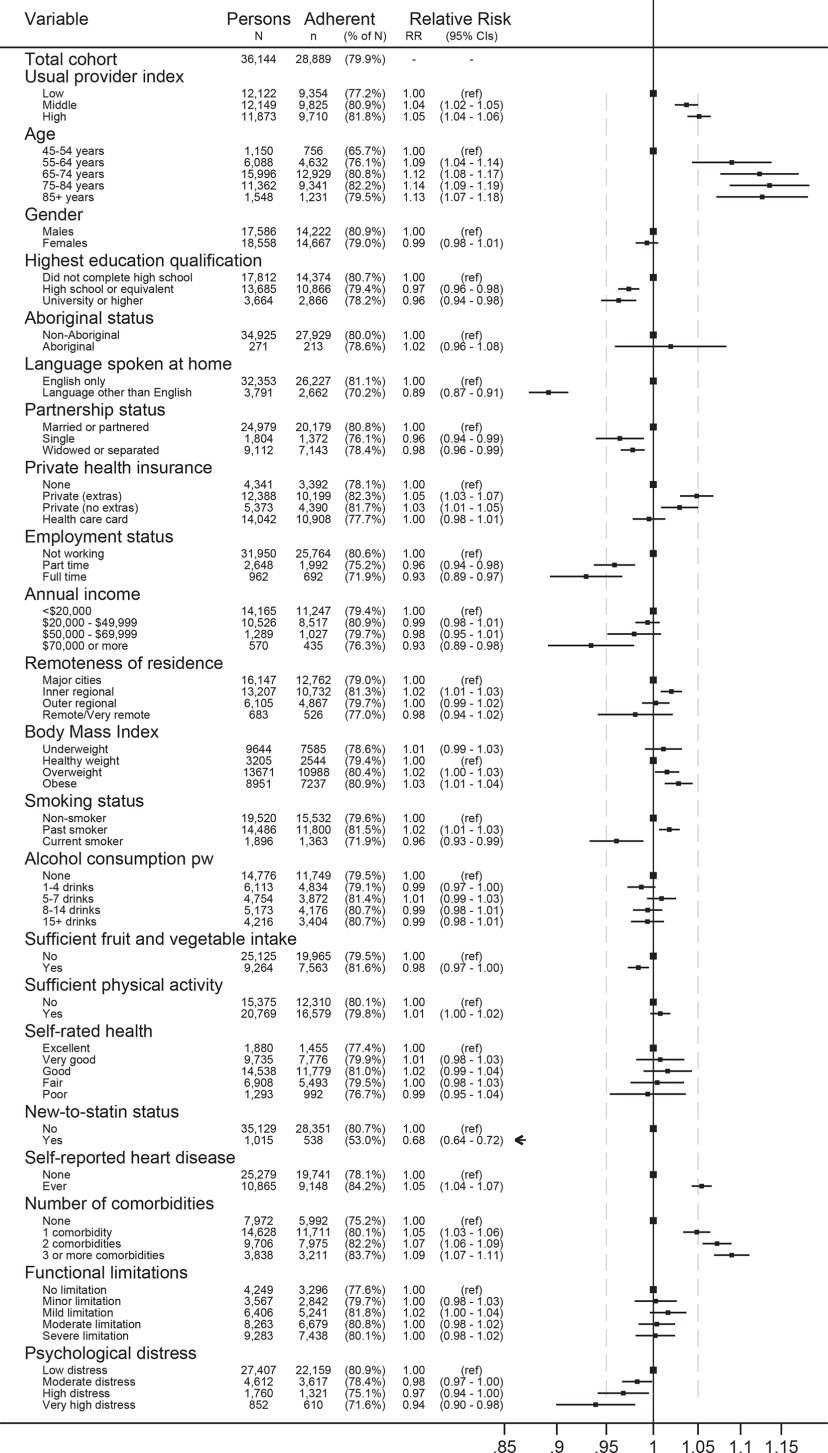
Relative risk for statin adherence (MPR ≥ 80%) of mutually adjusted covariates from model 2, with relation to the relative risk of the Usual Provider Continuity Index (UPI) for highest versus lowest tertiles.

Fig 2 shows the forest plot of adjusted RR and 95% CI from model 2 for tertiles of UPI and covariates, with track marks to highlight comparison to UPI for the other variables. A number of variables had RRs similar in magnitude to UPI. For lower adherence these were: university level education, being single, being employed, highest income band, current smoker and high to very high psychological distress. For higher adherence these were age 55–64 years, having private health insurance, history of heart disease and having one comorbidity. Being new to statins was the strongest predictor in the model (RR 0.68, 95% CI 0.64–0.72); age categories 65+, speaking a language other than English at home and having two or more comorbidities also had RRs clearly greater than that for UPI.

Only new-to-statins status and speaking a language other than English at home were found to have significant interaction with UPI on the association with MPR (p = 0.003 and p = 0.04, respectively). Stratification analyses by new-to-statins status showed a greater effect on adherence for those who were new to statins (model 2 RR 1.36, 95% CI 1.18–1.57 for medium versus low tertiles and RR 1.33, 95% CI 1.15–1.54 for high versus low tertiles) than people who were not new to statins (RR 1.03, 95% CI 1.02–1.04; RR 1.05, 95% CI 1.03–1.06 respectively). Similarly, model 2 analysis stratified by language other than English spoken at home showed that increased continuity had a greater impact among people who spoke a language other than English at home (model 2 RR 1.07, 95% CI 1.01–1.13 and RR 1.12, 95% CI 1.06–1.18, in medium versus low and high versus low tertiles, respectively) as compared to people who spoke only English at home (RR 1.03, 95% CI 1.02–1,05 and RR 1.04, 95% CI 1.03–1.06), although to a lesser extent than in people who were new to statins.

Results of propensity matched analyses are shown in Table 3. In these analyses, higher levels of UPI, either in tertile categories or dichotomised cut-off, were significantly associated with higher MPR for statin adherence. The effect size for the association between UPI and MPR generated from propensity matched analysis was similar to that from Poisson regression although the confidence intervals were wider.

**Table 3:** Association between Usual Provider Continuity Index (UPI) and statin adherence (Medication Possession Ratio MPR ≥ 80) using propensity score matching^*^.

		MPR ≥ 80		RR (95% CI)		
Propensity match	UPI (min-max)	No	Yes	Crude	Model 1	Model 2
**Match 1**						
Low tertile	9.0–66.7	2529 (22.4)	8788 (77.6)	1.00	1.00	1.00
Medium tertile	66.8–88.9	2185 (19.3)	9132 (80.7)	**1.04 (1.01-1.07)**	**1.04 (1.01-1.07)**	**1.04 (1.01-1.07)**
**Match 2** †						
Low tertile	9.0–66.7	2181 (22.7)	7444 (77.3)	1.00	1.00	1.00
High tertile	89.0–100	1781 (18.5)	7844 (81.5)	**1.05 (1.02-1.09)**	**1.05 (1.02-1.09)**	**1.05 (1.02-1.09)**
**Match 3**						
UPI <0.75	9.0–74.9	3297 (21.9)	11742 (78.1)	1.00	1.00	1.00
UPI ≥0.75	75–100	2818 (18.7)	12221 (81.3)	**1.04 (1.02-1.07)**	**1.04 (1.01-1.07)**	**1.04 (1.01-1.07)**

* Separate propensity matches were performed for the usual provider continuity index between cohorts of (1) low and medium tertiles; (2) low and high tertiles; (3) having a usual provider of care (UPI ≥0.75) or not. Propensity matching was performed using all covariates described in Table 1, including age, gender, highest education qualification, Aboriginal or Torres Strait Islander status, language other than English spoken at home, partnership status, private health insurance, employment status, annual household income, remoteness of residence, body mass index, current smoking status, alcohol consumption, fruit and vegetable consumption, physical exercise, self-rated health, self-reported heart disease, number of comorbidities, functional limitation, psychological distress, and new to statin status.

† There were no significant differences (Chi-square p-value < 0.05) between matched cohorts in the distribution of variables used for propensity matching, with the exception of physical activity and new to statin status within the cohort from Match 2.

## Discussion

We found that CoC was associated with greater adherence to statins. The magnitude and statistical significance of this association was similar regardless of the covariates that were adjusted for (age and sex only versus a wide range of socio-demographic and health related factors), modelling method (Poisson regression versus propensity score matching) and CoC measure used (UPI or CoC score). The findings are broadly consistent with a review that found CoC is associated with improved patient outcomes[36] and with a study of US veterans that found that those with three or more prescribers had lower refill adherence for dyslipidaemia medications.[37] Moreover, our findings suggest that the association of CoC with statin adherence is not simply a ‘healthy user bias’, since it was robust to adjustment for a range of healthy behaviours including smoking, alcohol intake, fruit and vegetable intake and physical activity. Although some health behaviour and health status variables were significantly associated with adherence (e.g. smoking status and prior heart disease), these did not appear to act as confounders or mediators of the CoC–adherence relationship, with the exception of new-to-statins status.

In keeping with the meta-analysis by Lemstra et al.,[11] we found that patients who were new to statins are at much greater risk of nonadherence. In addition, our stratified analysis demonstrated that the CoC association was much stronger for those who were new to statins. The findings are consistent with Brookhart et al.[14] with respect to positive association of CoC and statin adherence for new statin users. We extended these findings to longer-term statin users, and demonstrated the association to be largely invariant against socio-demographic and health related variables not available to the earlier study. Moreover, we found that the associations between CoC and statin adherence were similar when comparing the effects of the middle and upper tertiles versus the lowest tertile suggesting there was a threshold effect rather than a trend with increasing level of CoC.

Based on international findings, it is not surprising that we found patients in Australia speaking a language other than English at home to have lower statin adherence. In looking at cardiac medication use after acute myocardial infarction, Lai et al.[38] found some (although inconsistent) adherence risk for Chinese and South Asian groups compared to non-Asian Canadians. More consistent with our findings, Wisnivesky et al.[39] found limited English proficiency was associated with poorer self-management and worse outcomes among older people with asthma with respect to Hispanic American populations. Our analysis suggests that the association of CoC and statin adherence may be stronger for those speaking a language other than English at home. Further study of the mechanisms by which this group is associated with poorer adherence in the Australian context is warranted.

While the magnitude of the association we found between achieving higher tertiles of CoC and the likelihood of high statin adherence appeared to be modest, its effect size was similar to other recognised predictors of adherence, such as self-reported heart disease. The association of statin adherence and outcomes has been demonstrated in a number of contexts. For example, a longitudinal study among 31,455 elderly survivors in Ontario showed that low adherence to statins in the year after hospitalisation for myocardial infarction was associated with between 12–25% increased risk of mortality.[9] Further, a cohort study of patients newly treated with statins and initially free of cardiovascular disease showed that patients with low adherence to statins were more likely to be hospitalised and had increased hospital costs.[40] Therefore, even a small increase in likelihood of non-adherence could represent important information about opportunities for improved disease management.

Our dispensing data was limited to records of government subsidies (i.e. we did not have direct access to the data from pharmacies). As such, our analysis was restricted to participants who were Concession Card holders throughout the two-year period for which we measured adherence as only these participants had subsidy across the full range of statins during our analysis period. We had previously found that similar factors influence statin adherence in Concession Card holders and general beneficiaries.[12] Since our findings come from the Australian context, where there is a substantial degree of universal health subsidy, we expect financial factors to play a stronger role in other healthcare systems; indeed Lemstra et al.[11] found co-payment to be a significant factor for non-adherence, and found the reverse trend to the present study with lowest rather than highest income associated with non-adherence. Moreover, the Australian system allows health consumers relatively free choice of providers.

It is possible that statin users in our analysis are not entirely representative of the broader population. While 45 and Up Study participants had higher incomes, and lower prevalence of smoking, psychological distress, hypertension, diabetes and asthma than respondents in a population health survey, the prevalence of other characteristics such as body mass index and falls history was similar in the two studies.[41] We relied on self-reported data for some predictors, including socio-demographic and health-related variables; validation studies involving participants in the 45 and Up Study, however, have found excellent agreement between self-reported country of birth and that recorded in hospital data[42] and between body mass index categories from self-reported and measured data.[43] The very large size of the 45 and Up Study means that there is substantial heterogeneity within predictor variables, which is required for the valid estimation of relative measures of effect calculated, as here, from internal comparisons within a cohort.[44]

We calculated CoC measures at the practitioner level only and therefore did not consider the potential positive effect of continuity at the practice level that might come from use of practice-wide electronic medical records and recall systems. Unfortunately, Australian MBS data do not include a practice identifier. Moreover, we calculated CoC and medication possession concurrently in a 2-year time period. Thus, both factors could have been simultaneously influenced by unobserved variables (i.e. beyond those for which we adjusted in the analysis), and findings from this study might not be generalised for the association of COC and medication adherence that was measured in a lagged time period.

In summary, we found that CoC is associated with greater adherence to statins, particularly for patients who are new to statins. Our findings were observational and therefore do not imply that manipulating CoC, if feasible, will improve overall rates of adherence. However, they clearly indicate that when a patient has placed around 75% of their community care visits in a single provider, they more often remain adherent to statins, regardless of demographic, health status and behavioural factors. Our findings suggest that continuity-promoting practices–such as follow-up and other aspects of a strong patient-provider relationship–are promising for engendering better adherence to long-term medications and better CVD risk management.
